# False-positive imipenemase detected by NG-Test CARBA-5 in carbapenem-resistant *Acinetobacter baumannii*

**DOI:** 10.1128/spectrum.03757-23

**Published:** 2023-12-11

**Authors:** Emily Bentley, Carmella Russo, Ayesha Khan, Shanna Smalc, Daniel D. Rhoads, Romney Humphries, Lili Tao

**Affiliations:** 1 Department of Pathology, Microbiology and Immunology, Division of Laboratory Medicine, Vanderbilt University Medical Center, Nashville, Tennessee, USA; 2 Department of Laboratory Medicine, Cleveland Clinic, Cleveland, Ohio, USA; 3 Department of Pathology, Cleveland Clinic Lerner College of Medicine, Case Western Reserve University, Cleveland, Ohio, USA; 4 Infection Biology Program, Lerner Research Institute, Cleveland Clinic, Cleveland, Ohio, USA; University of California, San Diego, La Jolla, California, USA

**Keywords:** *Acinetobacter baumannii*, carbapenemase, false-positive reaction

## LETTER

*Acinetobacter baumannii* is a growing global health concern due to its ability to cause severe diseases including pneumonia and bacteremia, particularly in patients with extensive healthcare exposure (1, 2). *A. baumannii* is known to be persistent in harsh environments and causes healthcare-associated infections. Although carbapenems have remained as the most effective antibiotics for treating infections caused by *A. baumannii*, carbapenem-resistant *A. baumannii* (CRAB) has become prevalent worldwide, with resistance to meropenem ranging from 36.5% in North America to 60.5% in Europe, as reported by the SENTRY Antimicrobial Surveillance Program (3, 4). The emergence of carbapenem resistance in *A. baumannii* further increased disease morbidity and mortality and caused a significant economic burden (5 – 7).

Multiple mechanisms contribute to the development of carbapenem resistance in *A. baumannii*. These include production of carbapenemase enzymes, loss of porins, overexpression of efflux pumps, and alterations in penicillin-binding proteins. Production of carbapenemases is considered as the primary mechanism responsible for the development of carbapenem resistance in *A. baumannii*. While that detecting carbapenemase in *A. baumannii* is generally not recommended due to the prevalence of class D carbapenemases, including OXA-23, OXA-24/40-like, OXA-51-like, and OXA-58 in the majority of CRAB that express a carbapenemase, metallo-β-lactamases (MBL), including imipenemase (IMP), New Delhi metallo-β-lactamase (NDM), and verona integron-mediated metallo-β-lactamse (VIM) are responsible for a small proportion of CRAB (4, 8). Sulbactam-durlobactam was recently approved for use by the U.S. FDA, and it is intended to be used to treat CRAB pneumonia (https://www.fda.gov/news-events/press-announcements/fda-approves-new-treatment-pneumonia-caused-certain-difficult-treat-bacteria); however, CRABs with MBL are known to be resistant to sulbactam-durlobactam (9). Rapidly identifying an MBL, such as IMP, in an *A. baumannii* isolate using an immunoassay would preclude the empiric use of the sulbactam-durlobactam.

The NG-Test CARBA-5 is a lateral flow immunochromatographic assay (LFA) that was approved for the rapid detection of five most common carbapenemases in *Enterobacterales* and *Pseudomonas aeruginosa*, namely the *Klebsiella pneumoniae* carbapenemase (KPC), OXA-48-type carbapenemase (OXA-48), VIM, IMP, and NDM. Given the ease of workflow, rapidity, and low cost, many laboratories have adopted this diagnostic device to rapidly characterize bacterial isolates. It would be desirable to expand the use of this LFA to include CRAB, despite it not detecting the common OXA carbapenemases expressed by CRAB. Excluding MBLs would be useful in identifying CRAB isolates likely to be susceptible to sulbactam-durlobactam. In our laboratory, we modified the test during a plasmid-borne outbreak of *bla*_VIM_-producing, carbapenem-resistant Gram-negative bacteria that included CRAB isolates expressing *bla*_VIM_. Surprisingly, several isolates of CRAB were tested positive for IMP by the CARBA-5 test. Following a similar finding of unexpected IMP in CRAB by a second institution, we evaluated the potential of the CARBA-5 to detect carbapenemase in CRAB.

Eleven (11) CRAB isolates from Vanderbilt University Medical Center (VUMC) and 13 from Cleveland Clinic (CC) were evaluated. Isolates were derived from routine cultures. Identification was confirmed using Biotyper matrix-assisted laser desorption/ionization-time of flight mass spectrometry (Bruker, Billerica, MA). All isolates included in this study were identified as *A. baumannii* but not other members of the *A. baumannii* complex. Initial antimicrobial susceptibility testing was performed using Phoenix PMIC306 panel on M50 instrument (BD, Franklin Lakes, NJ) at VUMC and Sensititer Gram Negative CMC1GNCC panel at CC (Thermo Fisher Scientific, Waltham, MA). Meropenem resistance was further confirmed using Etest (biomerieux, Marcy-l'Étoile, France). Isolates were shipped to VUMC for testing, and all isolates were subcultured with a meropenem disk in the first quadrant to ensure selection of the carbapenem-resistant phenotype.

**Fig 1:**
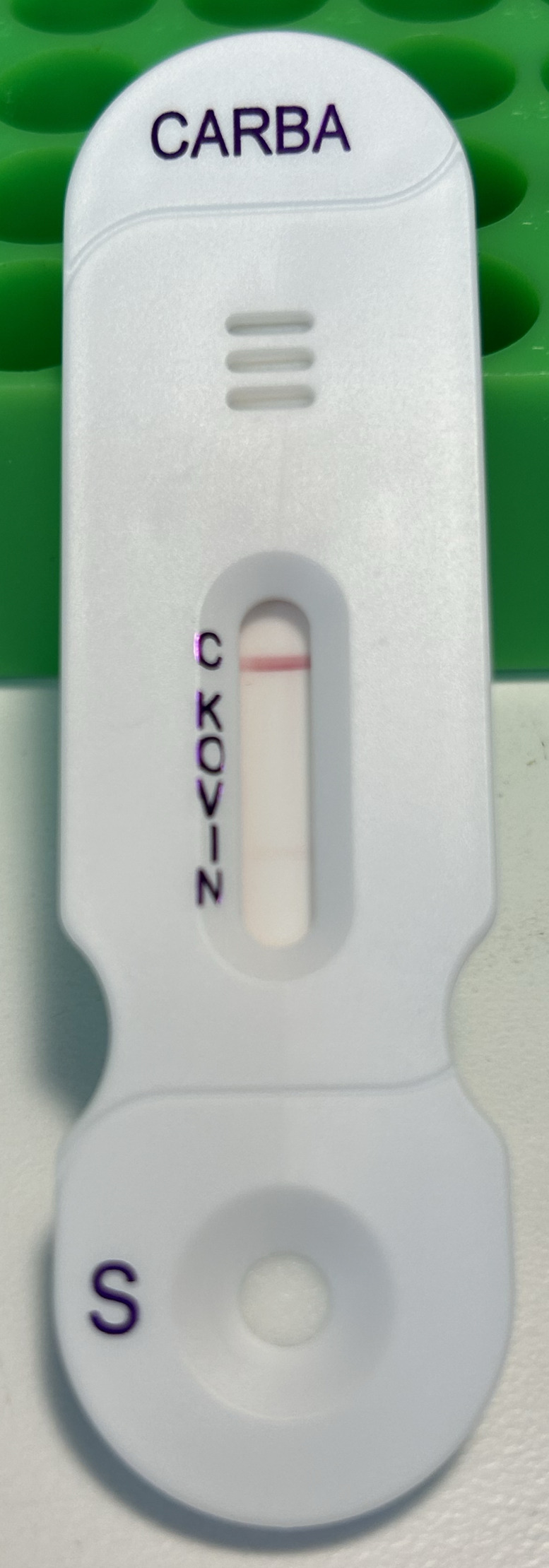
Positive IMP by NG CARBA-5 in carbapenem-resistant *Acinetobacter baumnnii*. The band appeared to be faint but readily visible.

NG-Test CARBA-5 lateral flow assay was performed according to the manufacturer’s instruction with the exception that *A. baumannii* was tested instead of *Enterobacterales* or *P. aeruginosa*. Three colonies from fresh culture were used to prepare bacterial lysate. Appearance of a line in any intensity was interpreted as a positive result. Of the 24 CRABs tested, 15 (62.5%) tested positive for IMP. One CRAB tested positive for both NDM and IMP, while another CRAB was tested positive for VIM but negative for IMP. All the positive IMP bands were faint but readily visible (Fig. 1).

The Cepheid Xpert Carba-R PCR was used to attempt confirmation of the presence of *bla*_IMP_ gene in the CARBA-5-positive CRAB isolates. The test was performed per the manufacturer’s instructions. All PCR tests were negative for all isolates.

MBL activity of the CRABs was evaluated phenotypically using a carbapenem-EDTA combination (10, 11). Specifically, MBL activity was evaluated using a carbapenem-EDTA disk method as previously described with modification (12). Meropenem-EDTA, imipenem-EDTA, and EDTA disks were prepared by adding 10 µL of 0.5 M EDTA (pH 8.0) to meropenem disk, imipenem disk, and blank filter disk, respectively, and the disks were air dried before use. Overnight incubated fresh bacterial colonies were diluted to 0.5 McFarland bacterial suspension in saline and inoculated to a Mueller-Hinton agar (MHA) plate. After drying, meropenem disk, imipenem disk, meropenem-EDTA, imipenem-EDTA, and EDTA disk were added to the inoculated plate. The MHA plates were incubated at 35°C in ambient air for 18–24 hours. A ≥7 mm increase in zone diameter with meropenem-EDTA and imipenem-EDTA disks compared to meropenem disk, imipenem disk, as well as EDTA disk is considered as screen positive for MBLs. Quality control strains *K. pneumoniae* BAA1705 (positive for KPC), *Escherichia coli* NCTC 13476 (positive for IMP), and *K. pneumoniae* BAA-2146 (positive for NDM) were included in each test. All controls yielded expected results, but none of the CRAB isolates demonstrated an increase in zone size with the addition of EDTA, indicating the absence of a functional MBL in these CRAB isolates, albeit this method is not well validated for *A. baumannii*.

To further investigate the possibility of false-positive IMP results by CARBA-5, we performed whole genome sequence (WGS) of the CRAB isolates. CRABs from CC were retested using a different batch of CARBA-5 by another operator prior to the WGS, yielding the the same results as the initial testing. Genomic DNA was extracted using the PureLink Genomic DNA Mini Kit (Invitrogen, Waltham, MA), and genomic libraries were prepared using NEBNext Ultra II DNA Library Prep Kit for Illumina (New England BioLabs, Ipswich, MA). The libraries were pooled, and WGS was performed using paired-end 150 bp on the Illumina NovaSeq 6000 (Illumina, San Diego, CA) with a target of 100 × coverage. Reads were assembled and annotated on online bacterial and viral bioinformatic center BV-BRC using the comprehensive genome analysis pipeline (https://www.bv-brc.org/app/ComprehensiveGenomeAnalysis). Antibiotic-resistant genes were identified according to The Comprehensive Antibiotic Resistance Database. The β-lactamases detected by WGS in these CRABs are listed in Table 1. All the CRABs harbored a *bla*_ADC_ class C β-lactamase and a *bla*_OXA-51-like_ class D carbapenemase. Additionally, most CRABs harbored either a *bla*_OXA-23_ or *bla*_OXA-24/40_ class D carbapenemases. Two CRAB isolates harbored an MBL, specifically *bla*_VIM_ and *bla*_NDM_, which mathed the VIM and NDM detected by CARBA-5. No *bla*_IMP_ gene were detected in any of the CRABs.

**TABLE 1:** Beta-lactamase genes identified using whole genome sequencing in the carbapenem-resistant *Acinetobacter baumannii* isolates included in the study

Strain#	IMP by CARBA-5	Beta-lactamases^*a*^
1	Positive	*bla*_ADC-2_, *bla*_CTX-M-15_, *bla*_OXA-223_, *bla*_OXA-23_, *bla*_OXA-24_
2	Positive	*bla*_ADC-2_, *bla*_OXA-24_, *bla*_OXA-66_
3	Positive	*bla*_ADC-2_, *bla*_OXA-24_, *bla*_OXA-338_
4	Positive	*bla*_ADC-2_, *bla*_OXA-24_, *bla*_OXA-338_
5	Positive	*bla*_ADC-2_, *bla*_OXA-24_, *bla*_OXA-66_
6	Positive	*bla*_ADC-2_, *bla*_CTX-M-15_, *bla*_OXA-24_, *bla*_OXA-66_
7	Positive	*bla*_ADC-2_, *bla*_OXA-23_, *bla*_OXA-24_, *bla*_OXA-66_
8	Negative	*bla*_ADC-2_, *bla*_OXA-24_, *bla*_OXA-66_
9	Negative	*bla*_ADC-2_, *bla*_OXA-23_, *bla*_OXA-66_
10	Negative	*bla*_ADC-2_, *bla*_CTX-M-15_, *bla*_OXA-24_, *bla*_OXA-66_
11	Positive	*bla*_ADC-2_, *bla*_OXA-23_, *bla*_OXA-82_
12	Positive	*bla*_ADC-2_, *bla*_OXA-23_, *bla*_OXA-66_
13	Positive	*bla*_ADC-2_, *bla*_OXA-24_, *bla*_OXA-95_
14	Positive	*bla*_ADC-2_, *bla*_OXA-23_, *bla*_OXA-66_, *bla*_TEM-1_
15	Positive	*bla*_ADC-2_, *bla*_OXA-24_, *bla*_OXA-95_
16	Positive	*bla*_ADC-2_, *bla*_OXA-82_
17	Positive	*bla*_ADC-2_, *bla*_OXA-82_
18	Negative	*bla*_ADC-2_, *bla*_OXA-24_, *bla*_OXA-95_
19	Negative	*bla*_ADC-2_, *bla*_OXA-23_, *bla*_OXA-24_, *bla*_OXA-95_
20	Negative	*bla*_ADC-2_, *bla*_OXA-82_
21	Negative	*bla*_ADC-2_, *bla*_OXA-23_, *bla*_OXA-66_, *bla*_TEM-1_
22	Negative	*bla*_ADC-2_, *bla*_OXA-24_, *bla*_OXA-95_
23	Positive	*bla*_ADC-30_, *bla*_NDM-1_, *bla*_OXA-23_, *bla*_OXA-66_
24	Negative	*bla*_ADC-26_, *bla*_VIM-1_, *bla*_OXA-64_, *bla*_TEM-1_

^*a*^ *bla*_OXA-64_, *bla*_OXA-66_, *bla*_OXA-82_, *bla*_OXA-95_, *bla*_OXA-223_, and *bla*_OXA-338_ all belong to the *bla*_OXA-51-like_ family.

Carbapenemase resistance in *A. baumannii* is mainly caused by the presence of class D carbapenemases, such as OXA-23 and OXA-24/40 and OXA-51-like family. However, class B metallo-β-lactamase enzymes, including IMP, VIM, NDM, and Seoul imipenemase, have been detected in the *Acinetobacter* spp. (4, 13). Identification of carbapenemase in CRAB may be implicated for infection control and treatment, as the new antimicrobials, including sulbactam/durlobactam, are not effective against MBL-producing *Acinetobacter* (14, 15). However, carbapenemase detection in CRAB is challenging for clinical laboratories. The modified carbapenem inactivation method and EDTA carbapenem inactivation method tests are currently not recommended for the detection of carbapenemase activity in *A. baumannii* (16). The CIMTris test has shown good performance in detecting carbapenemase phenotypically in *Acinetobacter* and *Pseudomonas* in a research setting, but its full evaluation in clinical testing is yet to be conducted (17, 18). The NG-Test CARBA-5 is not cleared by the U.S. FDA for the detection of carbapenemases in *A. baumannii* and the Xpert Carba-R PCR, while FDA cleared for use with *A. baumannii* can detect MBLs but not the more commonly encountered *bla*_OXA23_ and *bla*_OXA24/40_.

During a recent outbreak of a plasmid-borne *bla*_VIM_ expressing Gram-negative bacteria at our institution, CARBA-5 was considered as a convenient, rapid, and inexpensive method to rapidly screen for carbapenemases (and importantly VIM), based on its excellent performance with *Enterobacterales* and *Pseudomonas aeruginosa* (19 – 21) and anecdotes from colleagues and publications that showed successful use of the CARBA-5 with *A. baumannii* (22). Unexpectedly, we identified isolates positive for IMP during this assessment, which led to confusion during our outbreak investigation.

In the present study, we identified that 15 out of 24 CRABs yielded a false-positive IMP using the CARBA-5. The false-positive IMP usually appeared as a faint band, as shown in Fig. 1. However, the manufacturer’s product insert indicates that any band, regardless of the intensity, should be interpreted as positive. Coincidentally, a separate recent study also reported false-positive IMP in CRABs detected by CARBA-5 (17). In their initial testing, 93 out of 97 (95.9%) CRABs showed false-positive IMP by CARBA-5. Upon retesting, 61 out of 97 (62.9%) CRABs continued to generate false-positive results. The higher false-positive rate in their initial testing might contribute to the heavier inoculum used (six to seven colonies, as opposed to the three colonies recommended by the manufacturer). We adhered to the manufacturer’s recommended inoculum intensity and observed a similar false-positive rate for IMP as in their retesting.

In order to identify the factor that is responsible for the cross reaction to IMP with CARBA-5 test in CRABs, we conducted β-lactam resistome analysis using WGS data. However, no *bla*_IMP_ or possible *bla*_IMP_ variant was identified in all CRABs. Interestingly, we identified PA0057, an MBL superfamily protein gene in all the CRABs. It is unlikely that PA0057 is responsible for the IMP cross reaction in CRAB since it is present in all the isolates. Further research is necessary to identify the factor causing the false-positive reaction.

In conclusion, we detected high rate of false-positive IMP results in CRAB isolates using CARBA-5 test. Using PCR, a phenotypic MBL activity assay, and WGS, we were unable to corroborate the presence of IMP. We conclude that these IMP detections were false-positive LFA reactions. Off-label use of the CARBA-5 assay can result in false-positive IMP results when testing CRAB. Like any other diagnostic testing, it is crucial to conduct clinical validation to ensure the accuracy and reliability of results for the intended use case.
